# Implementing a bundle of interventions to support older adults transitioning from hospital to residential aged care: a protocol for the process evaluation of the OPTIMAL stepped wedge cluster randomised controlled trial

**DOI:** 10.1136/bmjopen-2025-106443

**Published:** 2026-02-12

**Authors:** Rangika L Fernando, Maria Crotty, Maria C Inacio, Ishita Batta, Alice Bourke, John Forward, Chloe Furst, Craig Whitehead, Sandra Shaw, Luke Shepperd, Gillian Harvey

**Affiliations:** 1 Flinders University, College of Medicine and Public Health, Flinders Health and Medical Research Institute, Adelaide, South Australia, Australia; 2 Registry of Senior Australians (ROSA) Research Centre, South Australian Health and Medical Research Institute, Adelaide, South Australia, Australia; 3 Rehabilitation, Aged Care and Palliative Care, Southern Adelaide Local Health Network, SA Health, Bedford Park, South Australia, Australia; 4 Flinders University, College of Nursing and Health Sciences, Caring Futures Institute, Adelaide, South Australia, Australia; 5 Royal Adelaide Hospital, Central Adelaide Local Health Network, SA Health, Adelaide, South Australia, Australia; 6 Aged Care, Rehabilitation and Palliative Care, Northern Adelaide Local Health Network, Modbury, South Australia, Australia

**Keywords:** Aged, Implementation Science, Health Services for the Aged, GERIATRIC MEDICINE, Randomized Controlled Trial

## Abstract

**Introduction:**

The Optimising older People’s Transition from acute care Into residential aged care through Multidisciplinary Assessment and Liaison (OPTIMAL) trial is a multisite hybrid type II stepped wedge randomised controlled trial with an embedded process evaluation that aims to evaluate the effectiveness of implementing a bundle of evidence-based interventions to provide systematic support to older adults being discharged from hospital to residential aged care (RAC) homes for the first time. The trial is based on evidence from models of care used internationally to improve the quality of care transitions and addresses a need to provide evidence of transferability and effectiveness of these models in the Australian context. The embedded process evaluation will assess the acceptability, appropriateness, feasibility, adoption and fidelity of the OPTIMAL intervention, as well as the mechanisms of impact.

**Methods and analysis:**

The OPTIMAL trial will be implemented across the three metropolitan local health networks (LHNs) in South Australia. The process evaluation will be conducted in parallel with the main trial and is theoretically informed by the integrated Promoting Action on Research Implementation in Health Services (i-PARIHS) implementation framework, which theorises that the implementation success of OPTIMAL is determined by the facilitation of the intervention with the intended recipients in their inner and outer contextual setting. The process evaluation will employ a mixed methods approach. Qualitative and quantitative data will be collected through baseline context mapping of LHNs, interviews with key LHN and RAC stakeholders, online survey of clinical teams, fortnightly check-in forms, and activity logs and field notes maintained by the nurse facilitator in each LHN. Data will be mapped and reported based on the i-PARIHS framework.

**Ethics and dissemination:**

Ethical approval for the OPTIMAL trial was obtained from the Southern Adelaide Clinical Human Research Ethics Committee (approval 2023/HRE00111), and the relevant governance approvals were obtained for each participating LHN. Ethical approval includes a waiver of the requirement for consent for routinely collected patient data. Study findings will be disseminated via journal publications, presentations at conferences, stakeholder discussions, consumer forums and advocacy to key decision makers to support knowledge translation.

**Trial registration number:**

Australia New Zealand Clinical Trial Registry, ACTRN12624001008516, registered 20 August 2024.

**STRENGTHS AND LIMITATIONS OF THIS STUDY**

- The proposed process evaluation is informed by the integrated Promoting Action on Research Implementation in Health Services implementation framework and intervention logic model.
- The mixed methods process evaluation uses a range of data collection methods and sources, at multiple time points over implementation.
- The Optimising older People’s Transition from acute care Into residential aged care through Multidisciplinary Assessment and Liaison (OPTIMAL) trial will be implemented across three local health networks (LHNs); thus, local contextual variation will be considered as part of the process evaluation, providing valuable information to inform future transferability and scalability of the OPTIMAL intervention.
- The prospective nature of the process evaluation enables factors influencing implementation to be tracked over time (such as staffing resources and motivation, availability of postdischarge services, organisational priorities, changes in models of care and implementation of new services).
- A potential limitation of the study is that data are not collected directly from older people who are receiving the intervention, but are collected from LHN stakeholders and clinical teams, residential aged care providers and the OPTIMAL nurse facilitator, due to logistical feasibility and resource constraints.

## Introduction

 Older adults transitioning from hospital to residential aged care (RAC; or long-term care) homes often have multiple chronic conditions and complex care needs and require good continuity of care as they move between providers and settings.^1^ Suboptimally managed care transitions can increase the risk of inappropriate care, medication errors and preventable emergency department (ED) visits or readmissions.^1 2^ Australian data show that within the first 90 days of entry into RAC, 22.6% of older adults present to an ED and 18.0% have an unplanned hospitalisation.^3^ Interventions to improve the quality of care transitions can reduce readmissions in older adults.^4 5^ Care transitions involve multiple disciplines and providers, but research demonstrates the pivotal role played by professional nurses in transitional care interventions.^6^

Quality of care transitions can be improved by interventions addressing one or more of the following: early discharge planning; communication of information in a timely, well-organised and accessible manner; medication safety; promoting self-management; enlisting the help of social and community supports; advance care planning; coordinating care across providers and settings; monitoring and managing symptoms after discharge; and timely follow-up after discharge.^4^,^10^ Several such models of care have been developed internationally to improve the quality of care transitions,^11^,^19^ but there is a need for evidence on the transferability and effectiveness of such models to improve care for older adults transitioning from hospital to RAC for the first time, in the Australian context.

In Australia, aged care and healthcare sectors have distinct regulatory and funding mechanisms and delivery models, and lack of integration between sectors can affect communication and collaboration during transitions between hospital and RAC.^20^ RAC refers to long-term accommodation and care (including both personal care and access to nursing and general health services) provided for older adults who require a higher level of support than can be offered in their own homes. RAC is regulated nationally by the Aged Care Quality and Safety Commission, and services are subsidised by the federal government through payments to approved providers (for-profit, not-for-profit or government-operated).^21^ In contrast, public hospitals are cofunded by federal, state and territory governments, but managed by state and territory governments through local health networks (LHNs).^22^ LHNs manage delivery of public hospital services and other community-based health services, usually within a defined geographical area, and are directly responsible for hospital performance. In 2023, there were 128 LHNs in Australia (including 10 in South Australia), and these greatly vary in their size, location and type of hospital (which can include both public and private hospitals).^23^ This separation of responsibilities between the federal government and state and territory governments can pose challenges to ensure consistent access to healthcare once in RAC.^20^ In view of the complexity of this environment, it is important to understand the transferability of the available evidence to the Australian setting.

The Optimising older People’s Transition from acute care Into residential aged care through Multidisciplinary Assessment and Liaison (OPTIMAL) study aims to implement a bundle of evidence-based interventions to provide systematic support for older adults being discharged from hospital to RAC for the first time and evaluate the effectiveness in terms of a reduction in avoidable ED presentations and readmissions. Details of the OPTIMAL trial, including the trial design, eligibility criteria, sample size calculation and participant recruitment, are described in a separate trial protocol paper,^24^ and the protocol for the embedded process evaluation is described here.

OPTIMAL will be implemented across the three metropolitan LHNs in South Australia (catering to approximately two-thirds of older South Australians), and the discharging hospital will be responsible for intervention delivery. The trial will enrol 1545 participants over 14 months, across the three LHNs. The OPTIMAL intervention draws on evidence that favours the use of complex, multicomponent interventions spanning the predischarge and postdischarge periods, and targeting patients at high risk of readmissions, in order to reduce hospital readmissions in older adults.^5 9 10^ Intervention complexity can arise from several interacting intervention components implemented across several providers, settings, health disciplines and organisation levels. In randomised controlled trials of complex interventions, in addition to evaluating the effectiveness, it is also important to understand how or why the intervention worked (or didn’t work), to what extent it was implemented as intended, the cost-effectiveness, and the scalability and transferability to other contexts.^25 26^ This is the purpose of the process evaluation protocol reported here.

Development of the intervention was theoretically informed by the integrated Promoting Action on Research Implementation in Health Services (i-PARIHS) framework, which proposes that implementation success is determined by the facilitation of an innovation (intervention) with the intended recipients in their inner and outer contextual setting.^27 28^

### The OPTIMAL intervention

The OPTIMAL intervention identifies older persons being discharged to an RAC home for the first time and stratifies them based on their risk of readmission through a data dashboard visible to clinicians. Risk categorisation uses an algorithm adapted from a model developed by members of the research team in a previous study, which predicts the risk of hospitalisations for older adults entering RAC.^3^ Based on the person’s risk category, the clinical team delivers a customised bundle of interventions to provide enhanced care during the transition to RAC. The effectiveness of the intervention will be assessed based on evidence of ED presentations and readmissions in the 30- and 90-day period following discharge, obtained from administrative data.

As noted, the OPTIMAL intervention is complex, comprising four core components with adaptable elements for each component (table 1). This allows flexibility to tailor the intervention to suit the local context, while maintaining the integrity of the core components.^25^ A locally appointed nurse facilitator plays a central role as an internal facilitator for implementation and works with the local team to tailor the intervention to suit the local context.^29^

**Table 1:** Components of the OPTIMAL intervention

	OPTIMAL core component	Adaptable element
1	Identification of older persons prior to being discharged to residential aged care for the first time.	None
2	Risk stratification of eligible patients based on their risk of readmission. Eligible patients and their risk categories are visible to the clinical team on the OPTIMAL data dashboard.	The number of risk categories can be decided by the local team: either two or three levels of risk (low/high or low/medium/high).
3	Delivery of a risk-stratified bundle of evidence-based interventions to provide enhanced care after discharge. Specific interventions and responsibility for delivery are to be decided by the local health network team based on the local context.	A bundle of interventions should include at a minimum A standard, same-day discharge summary for all patients. Case management by a registered nurse for those at high risk. Number and mix of interventions can be selected by the local team based on available resources and may include a postdischarge follow-up phone call from a nurse, medication reconciliation, access to a geriatric hotline and a postdischarge visit from a nurse.
4	Internal implementation facilitation by a locally appointed nurse facilitator for the duration of the intervention phase, with an additional 5 months to be distributed over the intervention establishment phase (minimum 1 month) and postintervention phase (minimum 3 months).	Options for role sharing and distribution of 0.6 FTE. A nurse facilitator may directly deliver the intervention and/or coordinate delivery from other services/units.

FTE, full-time equivalent; OPTIMAL, Optimising older People’s Transition from acute care Into residential aged care through Multidisciplinary Assessment and Liaison.

**Figure 1:**
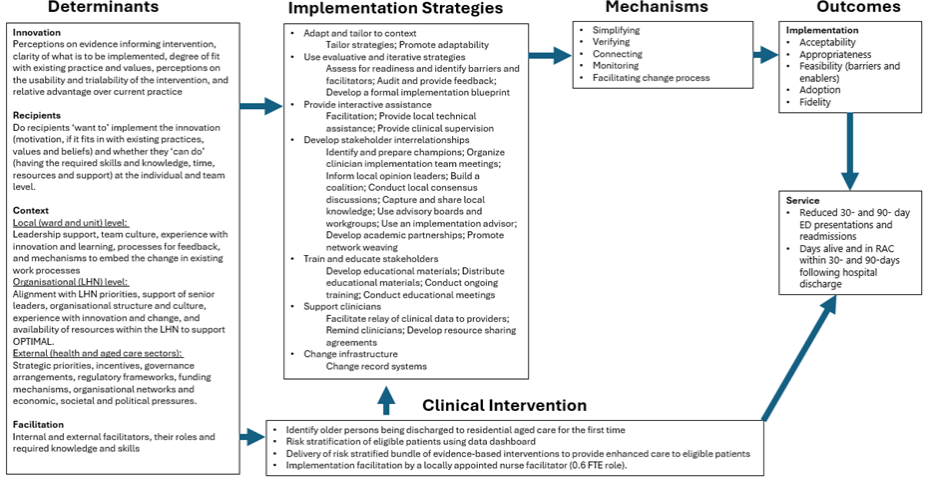
OPTIMAL intervention logic model.^30^ ED, emergency department; FTE, full-time equivalent; LHN, local health network; OPTIMAL, Optimising older People’s Transition from acute care Into residential aged care through Multidisciplinary Assessment and Liaison; RAC, residential aged care.

The research team held initial stakeholder discussions to define the scope of the problem, identify factors affecting a successful care transition and the currently available services. The stakeholders included: administrators and clinicians from participating LHNs, and representatives from RAC, ambulance services, primary care, consumers and researchers. This informed the development of an implementation research logic model for OPTIMAL (figure 1).^30^ The logic model describes the intended relationships between the determinants, strategies, mechanisms and outcomes as identified during the planning phase and will be used to track changes to implementation strategies over the course of the intervention, considering the complex and dynamic nature of implementation.^30^

### Implementation of the OPTIMAL intervention

The determinants of implementation are described according to the core elements of the i-PARIHS framework (ie, innovation, recipients, context and facilitation),^28^ and will be updated with information from the baseline context mapping. Based on these determinants, the OPTIMAL intervention will use the following categories of implementation strategies (as mapped against the Expert Recommendations for Implementing Change taxonomy) to achieve the intended outcomes: adapt and tailor to context, use evaluative and iterative strategies, provide interactive assistance, develop stakeholder interrelationships, train and educate stakeholders, support clinicians, and change infrastructure.^31 32^

The OPTIMAL intervention is intended to act through the mechanisms of *simplifying* the care transition process, *verifying* essential steps and checking for potential errors in the transition process; *connecting* healthcare providers in hospital, RAC and primary care settings; and *monitoring* participants to ensure that changes in their clinical status during the immediate period following the transition are noted and timely action is taken.^33^ The expected effects of the intervention encompass health service (ED presentations, readmissions and deaths within 30- and 90-day periods of discharge), and implementation outcomes (acceptability, adoption, appropriateness, feasibility, fidelity and implementation cost).^34^

### Process evaluation of the OPTIMAL trial

Guided by the UK Medical Research Council guidance on undertaking process evaluation studies, and in line with the hybrid type 2 study design, the OPTIMAL process evaluation seeks to understand an^25 35^:

1. Effectiveness perspective: to what extent does the OPTIMAL intervention achieve the intended implementation outcomes?
2. Theory-based perspective: how does the implementation effectiveness of the OPTIMAL intervention vary across different contexts, and why?

The process evaluation will support understanding of how and why the intervention works across different contexts, providing important information on transferability in the Australian context for decision makers. This will be useful to understand *what worked and how this can be optimised*, or *what failed, and why*.^35^ The study will incorporate the core elements of developing and evaluating complex interventions by considering the context of implementation, developing and refining the programme theory (as described previously and outlined in figure 1), engaging relevant stakeholders, identifying key uncertainties and conducting an economic evaluation.^25^ Although the core components of the OPTIMAL intervention (table 1) will not change over the course of the study, the intervention may be refined to respond to context and system changes.^25^

### Aims

The aim of the process evaluation is to assess the implementation of the OPTIMAL intervention in the three participating LHNs to:

1. Assess the acceptability, appropriateness and feasibility of the OPTIMAL intervention from the perspective of LHN and RAC stakeholders.
2. Assess the adoption and fidelity of the OPTIMAL intervention.
3. Evaluate whether the mechanisms of impact functioned as expected.

## Methods and analysis

### Study design

The study will use a theory-based perspective to conduct an embedded mixed-methods process evaluation, theoretically informed by the constructs of the i-PARIHS framework.

#### Innovation

i-PARIHS proposes that successful implementation is determined in part by support for the innovation, in this case, the OPTIMAL intervention. To assess this, the process evaluation will focus on the acceptability, appropriateness and feasibility of the OPTIMAL intervention to key LHN and RAC stakeholders due to their key role in delivering and receiving the intervention. It will assess their perceptions on the evidence informing the intervention, the clarity of what is to be implemented, and the degree of fit with existing practice and values. It will also assess perceptions on the usability and trialability of the intervention components (including risk stratification of patients and the data dashboard), and relative advantage over current practice.

#### Recipients

The recipient construct of i-PARIHS suggests that implementation success is determined by the motivation of the recipients of the innovation and their ability to implement the required change. LHN and RAC staff are the main recipients and it is important to understand whether they ‘want to’ implement the innovation (in terms of their motivation, if it fits in with existing practices, values and beliefs of the individual and their teams) and whether they ‘can do’ (in terms of whether they have the required skills and knowledge, time, resources and support at the individual and team level).

#### Context

i-PARIHS conceptualisation of contextual factors that influence implementation includes both inner and outer contexts and associated enablers and barriers to implementation. The inner context encompasses the local (ward and unit) level, and the organisation (ie, the LHN). Factors at the ward and unit level include leadership support, team culture, experience with innovation and learning, processes for feedback, and mechanisms to embed the change in existing work processes. Organisational factors affecting implementation include alignment with LHN priorities, support of senior leaders, organisational structure and culture, experience with innovation and change, and availability of resources within the LHN to support OPTIMAL.

The outer context refers to the health and aged care sectors at a state and national level in Australia, where implementation success may depend on their strategic priorities, incentives, governance arrangements, regulatory frameworks, funding mechanisms, organisational networks and economic, societal and political pressures that could affect the stability of the environment. RAC providers are diverse, and homes vary in size, location and ownership (government, for-profit and not-for-profit providers). The RAC context will be explored, to include the local and organisational level factors that may influence successful implementation. Engaging with the outer context through strategic communication and active networking can influence the penetration and sustainability of the innovation.

#### Facilitation

The i-PARIHS framework describes facilitation as the active ingredient for successful implementation, and internal implementation facilitation by a locally appointed nurse facilitator is one of the core components of the OPTIMAL intervention. The facilitator supports the adaptability of the intervention and, through their understanding and responsiveness to characteristics of the innovation, recipients and context/s that can present barriers and/or enablers of implementation. The facilitator will be supported by an OPTIMAL team in each LHN, and a central project team working closely with the LHN team as external facilitators to support implementation.

### Implementation outcomes

#### Acceptability, appropriateness and feasibility

Acceptability is the perception among key LHN and RAC stakeholders that the OPTIMAL intervention is satisfactory, based on their knowledge or experience of the content and complexity of the intervention.^36^ Acceptability can be dynamic and is based on their experience; thus, it will be assessed among LHN stakeholders during the pre-implementation and post implementation phases. Appropriateness refers to the perceived compatibility of the OPTIMAL intervention to the local setting, including providers, consumers and the problem to be addressed.^36^ This includes how well the intervention fits with the provider’s goals and service mandate, as well as how relevant it is to patients being discharged to permanent RAC for the first time in the South Australian setting. Feasibility is the extent to which the OPTIMAL intervention can be successfully delivered in the study wards, units and LHNs, as well as the receiving RAC homes, and relates to barriers and enablers to implementation, such as the availability of resources and required training.^36^

#### Adoption and fidelity

Adoption is the intention or decision to implement the OPTIMAL intervention and is measured from the perspective of the provider or organisation.^36^

Fidelity is the degree to which the OPTIMAL intervention is implemented as expected and will be assessed in terms of the content and dose (coverage, frequency and duration) delivered to participants.^36 37^ This includes conduct and attendance at LHN working group meetings, number and participation in training sessions by ward staff, and proportion of participants who receive the intended bundle of interventions (as per their risk category).

#### Functioning of mechanisms of impact as expected

As outlined in the logic model, a multicomponent implementation strategy will be employed. This is expected to generate the expected outcomes through mechanisms such as simplifying the care transition process, verifying essential steps, connecting providers and monitoring the clinical status (figure 1).^33^ The process evaluation will assess whether the mechanisms of impact have functioned as expected.

The process evaluation will employ a case study approach to describe the real-world context in which the OPTIMAL intervention is implemented in the three participating LHNs and explore causal factors for any differences in implementation and effectiveness. The researchers collecting and analysing qualitative data have medical/nursing backgrounds but are not undertaking any clinical roles during this research and are not employed by the LHNs (and therefore do not supervise or have authority over participants). They will maintain reflexivity during the study by appraising, recording and discussing established assumptions.

### Study setting and timeline

The process evaluation will be conducted in parallel with the main stepped wedge trial.^24^ The OPTIMAL intervention will be rolled out over 14 months, commencing 1 September 2024, across selected acute and subacute wards of hospitals in three metropolitan LHNs in South Australia. Each LHN is randomised to either the control or intervention phase, with one LHN per randomisation. Interventions start every 4 months, and all selected wards within an LHN commence the intervention together.

**Figure 2:**
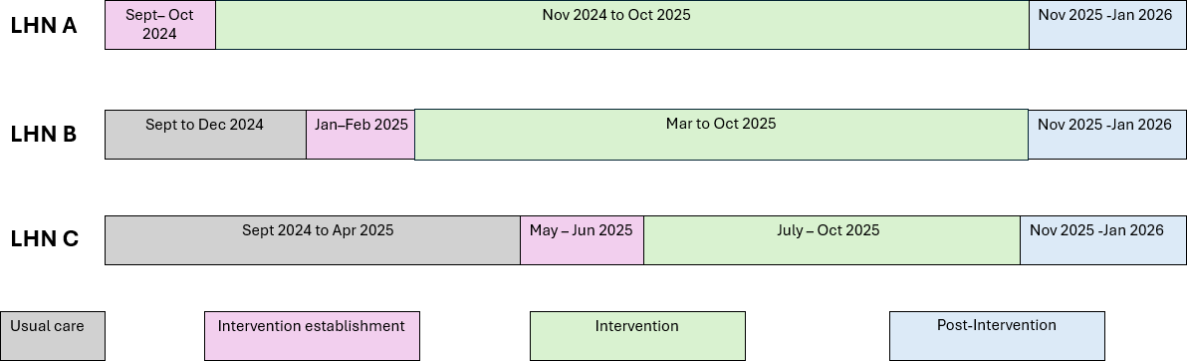
OPTIMAL study timeline. LHN, Local Health Network; OPTIMAL, Optimising older People’s Transition from acute care Into residential aged care through Multidisciplinary Assessment and Liaison.

The process evaluation will be conducted over the same 14 months, and for up to 3 months postintervention (ie, 1 September 2024 to 31 January 2026). It will include both quantitative and qualitative data collection, and participants will include administrators and clinical teams from participating units and wards in each LHN and staff of RAC homes receiving the discharged OPTIMAL participants. The timeline is shown in figure 2.

### Data collection

The acceptability, appropriateness and feasibility of the OPTIMAL intervention will be assessed through qualitative interviews with key LHN and RAC stakeholders, surveys of clinical teams and baseline context mapping. The adoption of the OPTIMAL intervention by the participating wards will be assessed using an OPTIMAL ward checklist and the OPTIMAL dashboard. Fidelity will be assessed through an activity log, a fortnightly check-in form and field notes maintained by the nurse facilitator. The study will explore whether the mechanisms of impact functioned as expected through qualitative interviews with key stakeholders, the check-in form, the activity log and field notes maintained by the nurse facilitator.

#### Baseline context mapping of LHN

During the intervention establishment phase, a baseline context mapping exercise will be carried out for each LHN to understand the likely barriers and enablers to implementation. This will include mapping the characteristics of the area population, the organisation structure and staffing in the units, existing policies and guidelines relevant to the study, the current process and pathway of patients being discharged to permanent RAC, and services available after discharge to RAC.

#### Qualitative interviews with LHN staff

Qualitative interviews with key decision makers in each LHN will be conducted at two time-points: prior to implementation of OPTIMAL (which will support the baseline context mapping) and in the postintervention period to assess the acceptability, appropriateness and feasibility of the OPTIMAL intervention, as well as the mechanisms of impact, and enablers and barriers to implementation. Interviews will be conducted using guides based on the i-PARIHS framework (online supplemental file 1; please note that the initial draft of the postintervention interview guide was updated prior to use in the study, based on findings from the pre-implementation phase and the updated guide is provided), with questions related to the innovation, recipients, context and facilitation (in the postintervention interview). Participants from each LHN will be selected by purposive sampling, according to their relevance to the aims of this process evaluation, and will include senior nursing leaders linked to the participating wards, a nurse unit manager (NUM), a senior allied health member of a multidisciplinary team relevant to the study, a doctor and a nurse from across the participating wards, as well as the OPTIMAL nurse facilitator. The research team will email potential participants to invite them to take part in an interview. Participation will be voluntary, and written informed consent will be obtained prior to the interview. Interviews are expected to last up to 45 min and will be conducted by a trained researcher. Participants will be given the choice of being interviewed in person or via Microsoft Teams at a time convenient to them.

#### Qualitative interviews with RAC staff

Qualitative interviews with staff of selected RAC homes will be conducted to assess their perspectives on the acceptability, appropriateness and feasibility of the OPTIMAL intervention, and the mechanisms of impact. The interviews will take place in the intervention and postintervention phases. Each LHN will be requested to provide a list of RAC homes that OPTIMAL patients have been discharged to. A minimum of five RAC homes per LHN will be purposively selected to represent the diversity of homes that OPTIMAL participants are discharged to (in terms of RAC home size, ownership and location) within each LHN. The research team will contact the management and/or staff at selected RAC homes via email and telephone to invite them to take part in an interview. Interviews are expected to last up to 45 min and will be conducted by a trained researcher using an interview guide based on the i-PARIHS framework (online supplemental file 2). Participants will be given the choice of being interviewed in person or via Microsoft Teams, and interviews will be arranged at a time convenient to the participant. Participation by RAC staff will be voluntary, and written informed consent will be obtained prior to the interview.

#### Survey of the clinical team

The interviews will be supplemented by a short online survey intended for clinical managers and members of the clinical team in the participating wards. The survey questionnaire (online supplemental file 3) is based on the i-PARIHS framework and the previously developed 34-item Mobilising Implementation of i-PARIHS (Mi-PARIHS) facilitation planning tool,^38^ which aids identification of facilitators and barriers related to the innovation, recipient and context constructs of the i-PARIHS framework to support planning and evaluation of implementation strategies. The Mi-PARIHS tool was adapted to the OPTIMAL intervention and the implementation context. Content validity of the survey questionnaire was assessed and confirmed by a clinician working in the local context and an expert in implementation science. Survey participants will be asked to respond to 25 statements related to the OPTIMAL intervention, the participant and their team, and the hospital in which they work. Participants will be asked to record their response to each statement on a 5-point Likert scale (strongly disagree to strongly agree). The survey questionnaire also includes two open-ended questions to stimulate participants to reflect on barriers to implementation and how these could be overcome, and any additional comments regarding the intervention or implementation at the participant’s site. Clinical managers and members of the clinical teams in the selected sites will be invited to participate via flyers in common staff areas and by email. The link and QR code for the online survey will be shared to enable participants to complete the survey online at a time and place convenient to them. Participation will be voluntary, and responses are deidentified. Participants will be provided with the information form and requested to indicate their consent using the online form prior to commencing the survey. The entire survey is expected to take approximately 15 min to complete, and a minimum of 10 responses from each site is anticipated.

#### OPTIMAL check-in form

The fortnightly OPTIMAL check-in form will be used to obtain feedback from the nurse facilitator at each site on participant enrolment during the period, updates from participating wards, staff training sessions conducted and attended, key action points at the fortnightly OPTIMAL steering group meetings, any adaptations made to the intervention during the period, and any service changes in the ward/unit/LHN during the period. Quantitative data, including the number (and hours) of training sessions held for ward staff, and the number and attendance at LHN working group meetings, will be collected from the check-in form.

Adoption by participating wards will be assessed by the nurse facilitator using a 5-item OPTIMAL ward checklist, namely: (1) at least one OPTIMAL poster displayed on the participating ward; (2) NUM has access to the dashboard; (3) NUM has had OPTIMAL training; (4) one nurse on the ward, picked at random, knows what the OPTIMAL intervention is (ie, it involves patients being discharged to RAC facility for the first time; participants are identified using the discharge plan note; can list the interventions for low risk patients; knows there is an OPTIMAL nurse facilitator to contact for further information/if patients want to opt out) and (5) at least one discharge pack is ready.

#### Nurse activity log and field notes

The nurse facilitator in each LHN will maintain an activity log to support delivery of the intervention to OPTIMAL participants and keep track of the participant’s journey from participating wards until discharge from health services. It will also record if, and when, the intended interventions are delivered to participants (eg, the date of the postdischarge follow-up call, time taken for the call and follow-up actions taken). The activity log will be supplemented, where necessary, by field notes, also maintained by the nurse facilitator in each LHN.

The research methods and data collection methods that will be used to assess the implementation outcomes are summarised in table 2.

**Table 2:** Mapping of the implementation outcomes, research methods and data collection methods

Implementation outcome	Research methods	Data collection methods
Acceptability Appropriateness Feasibility	Qualitative Quantitative	Qualitative interviews with LHN stakeholders and RAC staff Survey questionnaire Baseline context mapping
Adoption	Quantitative Qualitative	OPTIMAL ward checklist Qualitative interviews with LHN stakeholders
Fidelity	Quantitative Qualitative	Nurse activity log Check-in form Field notes
Whether the mechanisms of impact functioned as expected	Qualitative	Qualitative interviews with LHN stakeholders and RAC staff Nurse activity log Check-in form Field notes

LHN, local health network; OPTIMAL, Optimising older People’s Transition from acute care Into residential aged care through Multidisciplinary Assessment and Liaison; RAC, residential aged care.

### Data analysis

The team performing the process evaluation will be separate from the outcome evaluation team, but both teams will work together to ensure that data from the process evaluation can be integrated into the final analysis of OPTIMAL trial outcomes.^29^ The quantitative and qualitative data analysis will build on and complement each other.^29^ Where possible, the initial analysis will be completed prior to outcomes of the trial data being shared, to minimise bias in interpretation.^29^ A post hoc analysis will be conducted once trial outcomes are available to understand and explain any variations.

#### Qualitative data

Interviews will be digitally recorded (with participants’ consent) and transcribed using Microsoft software. Transcripts will be checked for accuracy against the digital recording, deidentified and stored in a secure university server. After initial familiarisation with the transcribed data, the data will be coded deductively using NVivo (Version 14) software and a theory-driven approach to map and report data based on the i-PARIHS constructs of innovation, recipients, context and facilitation. Qualitative data will also be extracted from the check-in forms, survey questionnaires, baseline context mapping, activity log and field notes. Data will be read and coded independently by two researchers (RLF and CMN) using content analysis. Qualitative description analysis will be applied to inductively code data that does not map to the i-PARIHS framework.^39^ Codes will be compared and refined through discussion until consensus is reached and conceptually grouped to provide a descriptive summary.^39^

#### Quantitative data

Data related to the adoption and fidelity will be analysed in IBM SPSS Statistics for Windows Version 29.0 (IBM Corp., Armonk, NY, USA) and presented using descriptive statistics (frequencies and proportions for categorical data, and sum, median (and IQR), and mean (and SD) for continuous data). This will include the hours of training, median time to the first follow-up phone call and proportion of eligible participants receiving the intervention components as intended. The survey results will be summarised using frequencies and reporting the mode and median scores for each statement. Scores will be compared preintervention and postintervention. Adoption in participating wards will be summarised using frequencies of scores on the OPTIMAL ward checklist.

The standards for reporting qualitative research (SRQR) reporting guideline^40^ was followed when drafting this manuscript (SRQR checklist is given in online supplemental file 4).

### Ethics and dissemination

Ethical approval for the OPTIMAL trial was obtained from the Southern Adelaide Clinical Human Research Ethics Committee (approval 2023/HRE00111), and the relevant governance approvals were obtained for each participating LHN. Approval for any identified variations to the approved protocol will be sought from the approving ethics committee and governance officers. The trial is prospectively registered with the Australia New Zealand Clinical Trial Registry ACTRN12624001008516, registered 20 August 2024. The ethical approval includes a waiver of the requirement for consent for routinely collected patient data. Written informed consent will be obtained from each participant prior to commencing the interviews, and informed consent will be obtained from all survey participants using the Qualtrics platform. Data will be deidentified and stored in a secure university server. Study findings will be disseminated via journal publications, presentations at conferences, stakeholder discussions, consumer forums and advocacy to key decision makers to support knowledge translation.

### Patient and public involvement

Consumer representatives from participating LHNs took part in stakeholder discussions during the planning phase of the research and contributed to define the scope of the problem and factors affecting a successful care transition. Consumer representatives will also be invited to dissemination workshops and plain language summaries will be prepared to support dissemination of findings to consumers.

## Discussion

The OPTIMAL trial is a multisite hybrid type II stepped wedge randomised controlled trial with an embedded process evaluation. The trial is based on evidence from models of care used internationally to improve the quality of care transitions and addresses a need to provide evidence of transferability and effectiveness of these models in the Australian context. The trial will be implemented across three LHNs in South Australia and involves a diverse range of RAC providers that OPTIMAL participants will transition to. The process evaluation will provide valuable information to inform future transferability and scalability of the OPTIMAL intervention by considering local contextual variation between settings and key factors that act as determinants of implementation success (or failure).

Implementation in the hospital setting is complex. Anticipated challenges include difficulties in collecting complete and accurate data both for primary data and existing clinical data, difficulties in recruiting participants for the process evaluation (interviews and surveys), and the regulatory requirements and approvals over multiple settings.^41^ The diversity and distribution of RAC providers pose similar challenges. The study will address these by involving clinical staff in developing and piloting data collection tools such as the activity log and check-in forms, and using an online survey and individual interviews rather than focus groups to offer flexible timing to improve participation.^41^ The team performing the process evaluation will coordinate with the outcome evaluation team to integrate data on implementation into analysis of outcomes where possible, which will also help to avoid duplication of work and measurement burden for site teams during the trial.^29^ The potential for bias in the analysis of outcome data due to this is acknowledged; however, where possible, this will be mitigated by the analysis and reporting of process data before the trial outcomes are shared.^29^

The study design is informed by the i-PARIHS implementation framework,^27 28^ and a key strength is that the research questions, methods and data collection are clearly mapped to the i-PARIHS constructs to guide the current process evaluation and to provide direction for future evaluations.^26^ The process evaluation is prespecified and conducted in parallel with the main trial; it employs a mixed methods approach to address the specified objectives and uses several data collection methods.^42^ Changes to the intervention over time will be captured by data collection at multiple time points, and data from all participants will be collected on key process variables, with more in-depth data obtained from smaller, purposively selected samples of key LHN stakeholders.^29^

The process evaluation seeks to understand the process of implementing the OPTIMAL intervention, including any contextual facilitators and barriers, and their effects on the health service outcomes of 30- and 90-day ED presentations and readmissions. During the OPTIMAL trial, the process evaluation may directly contribute to achieving the desired effects of the intervention through implementation facilitation. Relevant findings that emerge during the ongoing analysis of data, such as those related to modifiable barriers and enablers to implementation, will be discussed within the central project team, and where necessary, shared as feedback to the locally appointed nurse facilitator to enhance their responsiveness to characteristics of the innovation, recipients and context during implementation.

It is anticipated that the process evaluation will contribute to a better understanding of the overall process of implementation, scalability and transferability of the transitional care intervention in both the Australian context and internationally. Future steps would explore implementation in more specific contexts, such as in rural and remote settings, and involve participants and their families and carers to a greater extent. Greater understanding of how to optimise care transitions from hospital to RAC has the potential to improve the experiences of older people and their families and reduce the health system burden of potentially preventable hospital presentations and admissions.

## Supplementary material

10.1136/bmjopen-2025-106443 online supplemental file 1

10.1136/bmjopen-2025-106443 online supplemental file 2

10.1136/bmjopen-2025-106443 online supplemental file 3

10.1136/bmjopen-2025-106443 online supplemental file 4
