# Supplementary material for: Implementing a bundle of interventions to support older adults transitioning from hospital to residential aged care: a protocol for the process evaluation of the OPTIMAL stepped wedge cluster randomised controlled trial
Source: BMJ Open. 2026 Feb 12;16(2):e106443. doi: 10.1136/bmjopen-2025-106443 (PMC12911669; doi:10.1136/bmjopen-2025-106443)
Supplement: online supplemental file 1 [file bmjopen-16-2-s001.pdf]

INTERVIEW GUIDE

Optimising older People's Transition from acute care Into residential aged care through Multidisciplinary Assessment and Liaison

Pre-implementation (Context Assessment) interview

Local Health Network: ____

Job description: _______________________________________

Thank you for your time today. I will start with a bit of background: this project aims to improve the pathway of care for older people being discharged from hospital. As part of the clinical team providing care to older patients who are transitioning from hospital to residential care we are keen to get your inputs prior to implementation.

The intervention consists of a post-discharge bundle of evidence-based interventions to support older adults (aged 65 and over) who are transitioning from hospital to residential aged care for the first time. The interventions will be customised based on the patient’s risk of readmission and includes (1) a post-discharge phone call within 48-72 hours, (2) medication reconciliation by a pharmacist within one week, (3) same-day discharge summaries, (4) access to a geriatric hotline, and (5) a visit from an advanced geriatric practice nurse within seven days. The nurse will collaborate with the relevant GP and train residential care staff to enhance their capabilities.

We are keen to understand the context in which this will be implemented at your hospital, and to gain useful insights from you which will help to adapt the intervention to this context.

Innovation

1. What is the current practice for people transitioning from the hospital to residential aged care?
2. How does the intervention align with current practices at the hospital(s)?
3. How is the intervention perceived?
    a. Is there a shared view about the evidence for this intervention?
    b. Trust/scepticism about the intervention?
4. How easy/difficult would it be to implement at this hospital?
    a. Are any adaptations or tailoring required to implement it?
    b. Do you feel that there will be sufficient time to build experience and reflect upon and test the intervention?
5. What are the potential benefits of this intervention?
    a. What advantages are there from implementing this intervention for hospital staff, patients, residential aged care facility staff?

Recipients (thinking about within your team)

6. Who would be involved in implementing the intervention?
    a. What are their roles?
7. What are the motivators for the clinical team to implement this intervention?

    a. What are the rewards and incentives?
    b. What are the disincentives?
8. What capacity is there within the team to implement the change?
    a. How would it change roles and responsibilities?
    b. What specific skills and knowledge will be needed?
9. What new/additional resources will be needed?
    a. Can these be provided?
10. What support will be there for staff?
    a. Key leaders/clinical champions/stakeholders
11. Will it be easy to coordinate across teams and staff?
    a. Collaboration and team cohesiveness
    b. How would information sharing occur between staff and teams?
    c. What are the possible barriers?
12. How would implementation of the intervention challenge work within the clinical team?
    a. Between teams?

Thinking about the local context within which your team works

13. What aspects of the staff culture supports innovation and change?
    a. Is there a culture of learning and innovation?
    b. How would you describe the collaboration and teamwork
    c. What can be done to facilitate communication and shared work processes
    d. What are the barriers in relation to team culture?
14. What sort of vision, support, motivation can be expected from leaders?
15. What is the experience with implementing innovations/change at a team level?
16. How does staff receive feedback on their work?
17. How could we get feedback from staff during the implementation process?
18. How would the team access the required information and training?
    a. What would be the best way to deliver this training?

Thinking about the organisational context

19. How does implementation of this intervention align with the goals and strategic priorities of the hospital?
20. What level of support is there from senior leaders/managers?
    a. Who are the key leaders whose support is required for successful implementation?
21. What approaches would support innovation and change at this hospital?
    a. What is the experience with implementing innovations/change at this hospital?
    b. What are the barriers to change?
    c. How can change be embedded/sustained?

Thinking about the external health system

22. What external influences would most affect successful implementation of the intervention?
    a. What external motivators and incentives exist?
    b. What would hinder implementation?
    c. What is the likely impact from regulatory frameworks?
23. What opportunities are there for engaging with and leveraging external support for implementation?

Other

24. Are there any other challenges you anticipate to implementation?
    a. How could these be overcome?
25. Is there anything else that you would like to talk about that we haven't touched on?

Thank you for your time!

INTERVIEW GUIDE

Optimising older People's Transition from acute care Into residential aged care through Multidisciplinary Assessment and Liaison (OPTIMAL)

Evaluation Interview

Local Health Network: ____

Job description and role in OPTIMAL: __________________

Thank you for your time today. I will start with a bit of background: the OPTIMAL project aims to improve the pathway of care for older people being discharged from hospital. As part of the clinical team providing care to older patients who are transitioning from hospital to residential care, we would like to get your inputs regarding the implementation of the intervention. We are keen to understand your experience with implementing the post-discharge bundle of interventions to support older adults transitioning from your hospital to residential aged care for the first time.

Thinking about the OPTIMAL intervention (innovation)

1. What was your experience with implementing the intervention?
   a. Were the components well defined, communicated and understood?
   b. How easy or difficult was it to implement?
   c. What worked well? What didn't? Why?
2. Was there a need for this intervention?
   a. How was it perceived? Did it fill a gap?
   b. Was there enough evidence to get leaders and team members on board?
3. Do you think it offered any advantages over existing practice? How?
   a. How do you think it supported the transition to residential aged care?
4. Did it fit well with existing practices and the local setting?
   a. Did it require significant changes in processes and practice?
   b. What adaptations were made during implementation?
   c. Would any further adaptations be required to better suit local needs?
5. Was there enough time to build experience, test the intervention and address any problems/inefficiencies?
6. What results did you observe? For staff, patients, carers, residential aged care providers,

Thinking about teams (recipients)

7. Who was involved in implementing the OPTIMAL intervention?
   a. What were their roles?
   b. Were they sufficiently engaged? What motivated them? Any incentives/disincentives?
   c. How were disincentives addressed?
8. How did staff responsibilities and tasks change?
   a. Did they have the required skills and knowledge?
   b. Were sufficient time, resources and support available?
   c. Did staff have the required authority for effective implementation?
9. Were there ward champions/opinion leaders? How did this influence implementation?

10. Was it easy to coordinate and communicate between staff and teams?
    a. How did information sharing occur between staff and teams?
    b. What were the barriers? How were they overcome?
11. Were boundaries encountered that affected implementation? How were these addressed?
    a. Between teams? Sites? With external stakeholders?

Thinking about the local context (division) within which your team works

12. Who were the formal and informal leaders involved in implementation?
    a. How did they influence implementation?
    b. How were staff supported to take on new roles if required?
13. Did the staff culture support implementation?
    a. Were there any barriers?
    b. Did staff feel actively involved in the change?
    c. How would you describe the collaboration and teamwork?
14. To what extent was the intervention embedded in existing work processes and staff responsibilities? What could have been done differently?
15. In what ways does the division support learning, feedback and evaluation (e.g.- regular team meetings, audit and feedback processes, professional development)
    a. Was there sufficient information and training on the intervention and implementation?
    b. How did staff access the required information and training?
    c. How did staff give and receive feedback during implementation?
    d. What sort of feedback was given/received?
    e. How was progress of implementation tracked?

Thinking about the organisational context (Local Health Network)

16. Did the intervention align with the goals and strategic priorities of the LHN?
    a. Was it seen as a priority for the LHN? If not, why?
17. How well were the goals of this project communicated, monitored and reported?
18. What supported/challenged implementation in this LHN? How were challenges addressed?
    a. Support from senior leaders
    b. LHN structure, systems and processes
    c. Organisation culture and capacity
    d. Opportunities for learning and feedback

Thinking about the external health system

19. Did the intervention align with strategic priorities for the wider health system?
20. Are there any incentives that reinforced this change? Eg – regulation, performance incentives
    a. Any disincentives?
21. What external influences affected (supported/challenged) implementation? How were challenges overcome?
    a. Societal pressures, new policies and services, critical events
22. Were there opportunities to engage with and leverage external support for implementation?
    a. Clinical networks, consumer groups
    b. Did networking between the LHN and residential aged care facilities change during implementation? How?

23. Do you think the change will be embedded/sustained?
    a. What components do you think might be sustained in practice?

Thinking about facilitation (for all interviewees, other than the OPTIMAL Nurse Facilitators)

24. Who were the internal facilitators and how did they support implementation?
    a. What was done to facilitate communication and shared work processes?
    b. Was any other support required? How could this have been provided?

Thinking about facilitation (for the OPTIMAL Nurse facilitators only)

25. Could you describe your role and responsibilities as the OPTIMAL Nurse Facilitator?
    a. What other roles do you hold within the LHN? How has this influenced your role as a facilitator?
    b. What were your main strategies and activities during implementation (establishment and intervention phases)
        - How did you engage staff and motivate staff?
        - How did you engage key (LHN and external) stakeholders?
        - How did you facilitate communication, feedback and shared work processes?
    c. What challenges did you encounter? How were these overcome?
    d. How were you supported in your role? By leaders, staff, other services, research team
    e. Did you have access to the required resources?
    f. What further support was required?

26. Is there anything else that you would like to talk about that we haven't touched on?

Thank you for your time!
