# Supplementary material for: Implementing a bundle of interventions to support older adults transitioning from hospital to residential aged care: a protocol for the process evaluation of the OPTIMAL stepped wedge cluster randomised controlled trial
Source: BMJ Open. 2026 Feb 12;16(2):e106443. doi: 10.1136/bmjopen-2025-106443 (PMC12911669; doi:10.1136/bmjopen-2025-106443)
Supplement: online supplemental file 2 [file bmjopen-16-2-s002.pdf]

Interview guide

Optimising older Peoples Transition from acute care Into residential aged care through Multidisciplinary Assessment and Liaison (OPTIMAL)

Interview: Residential Aged Care Homes

Thank you for your time today. This is part of a project which aims to improve the transition for older people being discharged from hospital to residential aged care for the first time.

We would like to hear your perspectives and experiences on the care for older people discharged from hospital to residential aged care (RAC) homes for the first time

1. What is the current process for taking over care of first time residents discharged from hospital to this RACF home?
   a. How is information received at discharge integrated into the residents' care plan at the RACF home?
2. What factors influence the decision making process for accepting placement of first time residents?
3. What is usually the difference in the care processes for residents taken in for respite and permanency?
4. In your experience, what does a good transition look like for someone being discharged from hospital to RACF for the first time?
   a. What supports a good transition?
   b. What is missing? What could be improved?
   c. What are the main challenges?
5. What are the main drivers for avoidable emergency department (ED) visits or readmissions within the first 3 months after discharge from hospital to for first time residents?
6. What supports or services would help to reduce avoidable ED visits or readmissions?

The OPTIMAL study is being implemented across selected wards in CALHN, SALHN, and NALHN. The study identifies older persons being discharged to a RACF home for the first time and predicts their risk of readmission as low, medium, or high prior to discharge. The LHN then provides a bundle of services (known as the OPTIMAL intervention) to support participants during their transition to RACF, and this is coordinated by an OPTIMAL project nurse in each LHN. The services provided depend on the risk level of the participant. As an example - a low risk participant could receive a discharge communication pack with their discharge summary, a follow-up call from the OPTIMAL nurse within 3 to 5 days after discharge, and access to a geriatric hotline, whereas those at high risk would receive more follow-up calls and visits (as required) from the OPTIMAL nurse.

Thinking about the OPTIMAL study,

7. Do you think there is a need for OPTIMAL?
    a. Would it improve transition to RACF? How?
8. How do you think OPTIMAL is/would be perceived by management and staff at this RACF?
9. What are the potential benefits for management, staff, residents, and their families?
10. To what extent does/would OPTIMAL fit with current practices at this RACF?
    a. How would it change current RACF practice?

Thinking about your RACF,

11. To what extent would/does OPTIMAL align with the goals and priorities of the RACF?
12. Who is/would be engaged in OPTIMAL from your RACF (eg – receiving and processing discharge information, receiving follow-up calls, communicating with the OPTIMAL nurse or hotline)?
    a. Would it change roles and responsibilities?
    b. What might motivate engagement in OPTIMAL?
    c. What might hinder engagement in OPTIMAL?
    d. What could be done to support/increase engagement?
13. How easy is it to communicate and coordinate between staff at your RACF?
    a. What is the best way to communicate information regarding care of a resident (such as discharge documentation), following discharge from hospital for the first time. Is it through email? Hard copies?
    b. What is the best way to communicate information about support services (such as the OPTIMAL nurse's contact number, outreach services)? Is it through email? Hard copies? Website? Webinar?
    c. How is/would information be shared with the relevant staff?
14. What is your experience with implementing innovation and change in this RACF?
15. Have you had any experience with residents on the OPTIMAL study? If yes, what is your experience with OPTIMAL?
    a. What is working well?
    b. What is not working well?
    c. What can be done to improve the interventions?
    d. Are you aware of a dedicated point of contact for OPTIMAL participants?
    e. Is this something you have used, or would use? Why?

Thinking about the role of the OPTIMAL nurse facilitator,

16. How well has the relationship with the OPTIMAL nurse worked in your experience?

   a. What is working well?
   b. What is not working well?
   c. How could it be improved?

Thinking about the health and aged care systems,

17. What external influences affect successful placement and transition of older people discharged from hospital to RACF?
    (Policies, regulations, information systems)
    a. What enhances successful placement and transition?
    b. What hinders successful placement and transition?
18. Are there opportunities to engage with other health and aged care stakeholders to improve outcomes for older people being discharged to RACF?

19. Is there anything else that you would like to talk about that we haven't touched on?

Thank you for your time!
