# Supplementary material for: Implementing a bundle of interventions to support older adults transitioning from hospital to residential aged care: a protocol for the process evaluation of the OPTIMAL stepped wedge cluster randomised controlled trial
Source: BMJ Open. 2026 Feb 12;16(2):e106443. doi: 10.1136/bmjopen-2025-106443 (PMC12911669; doi:10.1136/bmjopen-2025-106443)
Supplement: online supplemental file 3 [file bmjopen-16-2-s003.pdf]

Optimising older People's Transition from acute care Into residential aged care through Multidisciplinary Assessment and Liaison (OPTIMAL)

Survey questionnaire

Site/Hospital: Date:

Job description: Duration in this role:

The intervention consists of a post-discharge bundle of evidence-based strategies to support older adults (aged 65 and over) who are transitioning from hospital to residential aged care for the first time. The intervention will be customised based on the patient's risk of readmission and could include

(1) a standard, same-day discharge summary,

(2) a discharge wrap-around communication pack,

(3) a post-discharge phone call within 48-72 hours,

(4) access to a geriatric hotline,

(5) case management by a Nurse Consultant, and

(6) medication management review as required.

Please respond to the following statements about implementation of the intervention in your unit and hospital. For each statement, please tick the response that most closely reflects your view.

Statement	Strongly Disagree	Disagree	Unsure	Agree	Strongly Agree
**Thinking about the intervention (the post discharge bundle described above)**					
1. The evidence supporting this intervention is strong					
2. This intervention will improve patient outcomes (e.g.- reduce readmissions) and experience					
3. I understand what this intervention is aiming to achieve					
4. I understand what is needed to implement the intervention bundle					
5. This intervention needs to be adapted/tailored to this hospital					
6. This intervention offers an advantage over the current practice					
**Thinking about yourself and your team**					
7. My experience and available data reflect a need to improve the current practice for patients transitioning from hospitals to residential care					
8. I think this intervention is valuable and worthwhile implementing					

9. I have the necessary knowledge and skills to implement this intervention					
10. This intervention will require a significant change in daily routine					
11. I don’t have the workload or time capacity to implement this intervention					
12. My team and colleagues will be supportive to implement this intervention					
13. It will be easy to coordinate within the team to implement this intervention					
14. The existing infrastructure (I.e. equipment, IT, systems and processes) can support implementation					
15. My unit/hospital will provide the resources needed to support implementation					
**Thinking about the hospital in which you work**					
16. I feel actively involved in decisions that affect me					
17. My roles and responsibilities are clearly defined					
18. The culture supports innovation and change in practice					
19. I feel supported by team leaders/managers to carry out new initiatives/innovations					
20. There is good multi-disciplinary collaboration and teamwork					
21. I regularly receive feedback on my work					
22. I have previous experience in implementing innovations/change					
23. There is a culture of learning and innovation in this hospital					
24. This intervention aligns with the strategic priorities of my team/hospital					
25. External organisations/networks will be helpful to support implementation					

26. What barriers/challenges do you think there might be to implementing this intervention? How could these be overcome?

____
____
____
____

27. Do you have any other comments regarding this intervention or implementation at your hospital?

____
____

We thank you for your time spent taking this survey.

Your response has been recorded.
