# Supplementary material for: Implementing a bundle of interventions to support older adults transitioning from hospital to residential aged care: a protocol for the process evaluation of the OPTIMAL stepped wedge cluster randomised controlled trial
Source: BMJ Open. 2026 Feb 12;16(2):e106443. doi: 10.1136/bmjopen-2025-106443 (PMC12911669; doi:10.1136/bmjopen-2025-106443)
Supplement: online supplemental file 4 [file bmjopen-16-2-s004.docx]

**Supplementary File 4**

**The Standards for Reporting Qualitative Research (SRQR) reporting checklist for the manuscript ‘*Implementing a bundle of interventions to support older adults transitioning from hospital to residential aged care: a protocol for the process evaluation of the OPTIMAL stepped wedge cluster randomised controlled trial’.***

| **Number** | **Topic** | **Item Description** | **Location (or reasons for not reporting)** |
| --- | --- | --- | --- |
| **Title and Abstract** | | | |
| S1 | Title | Concise description of the nature and topic of the study identify the study as qualitative or indicating the approach or data collection methods. | Title (page 1)  This is a mixed methods study, as such the title does not identify the study as qualitative. |
|  | Abstract | Summary of key elements of the study using the abstract format of the intended publication. | Abstract (page 1-2) |
| **Introduction** | | | |
| S3 | Problem Formulation | Description and significance of the problem/phenomenon studied; review of relevant theory and empirical work; problem statement | Introduction (pages 4-9) |
| S4 | Purpose or research question | Purpose of the study and specific objectives or questions. | Introduction (pages 10-11) |
| **Methods** | | | |
| S5 | Qualitative approach and research paradigm | Qualitative approach and guiding theory if appropriate; identifying the research paradigm is also recommended; rationale | Methods (page 14-15) |
| S6 | Researcher characteristics and reflexivity | Researchers’ characteristics that may influence the research, including personal attributes, qualifications/experience, relationship with participants, assumptions, and/or presuppositions; potential or actual interaction between researchers’ characteristics and the research questions, approach, methods, results, and/or transferability | Methods (page 14-15) |
| S7 | Context | Setting/site and salient contextual factors; rationale | Introduction (page 4-6)  Methods (page 15) |
| S8 | Sampling strategy | How and why research participants, documents, or events were selected; criteria for deciding when no further sampling was necessary; rationale | Methods (pages 15-20) |
| S9 | Ethical issues pertaining to human subjects | Documentation of approval by appropriate ethics review board and participant consent, or explanation for lack thereof; other confidentiality and data security issues | Ethics and dissemination (pages 22-23) |
| S10 | Data collection methods | Types of data collected; details of data collection procedures including start and stop dates of data collection and analysis, iterative process, triangulation of sources/methods, and modification of procedures in response to evolving study findings; rationale | Methods (pages 15-20) |
| S11 | Data collection instruments and technologies | Description of instruments and devices used for data collection; if/how the instruments change over the course of the study | Methods (pages 15-20) |
| S12 | Units of study | Number and relevant characteristics of participants, documents, or events included in the study; level of participation | Methods (pages 15-20) |
| S13 | Data processing | Methods for processing data prior to and during analysis, including transcription, data entry, data management and security, verification of data integrity, data coding, and anonymization/deidentification of excerpts | Methods (pages 21-22) |
| S14 | Data analysis | Process by which inferences, themes, etc. were identified and developed, including the researchers involved in data analysis; usually references a specific paradigm approach; rationale | Methods (pages 21-22) |
| S15 | Techniques to enhance trustworthiness | Techniques to enhance trustworthiness and credibility of data analysis; rationale | Methods (pages 21-22) |
| **Results/findings** | | | |
| S16 | Synthesis and interpretation | Main findings; might include development of a theory or model, or integration with prior research or theory | n/a |
| S17 | Links to empirical data | Evidence to substantiate analytic findings | n/a |
| **Discussion** | | | |
| S18 | Integration with prior work, implications, transferability, and contributions to the field | Short summary of main findings; explanation of how findings and conclusions connect to, support, elaborate on, or challenge conclusions of earlier scholarship; discussion of scope of application/generalizability; identification of unique contributions to scholarship in a discipline or field | n/a |
| S19 | Limitations | Trustworthiness and limitations of findings | n/a |
| **Other** | | | |
| S20 | Conflicts of interest | Potential sources of influence or perceived influence on study conduct and conclusions; how these were managed | Competing interests statement (page 29) |
|  | Funding | Sources of funding and other support; role of funders in data collection, interpretation, and reporting | Funding statement (page 28) |

|  | Reference:  O'Brien BC, Harris IB, Beckman TJ, Reed DA, Cook DA. **Standards for reporting qualitative research: a synthesis of recommendations.** *Academic Medicine*, Vol. 89, No. 9 / Sept 2014 |
| --- | --- |
