# Supplementary material for: Implementing a bundle of interventions to support older adults transitioning from hospital to residential aged care: a protocol for the process evaluation of the OPTIMAL stepped wedge cluster randomised controlled trial
Source: BMJ Open. 2026 Feb 12;16(2):e106443. doi: 10.1136/bmjopen-2025-106443 (PMC12911669; doi:10.1136/bmjopen-2025-106443)
Supplement: Supplementary file 5 [file bmjopen-2025-106443.reviewer_comments.pdf]

PEER REVIEW HISTORY

BMJ Open publishes all reviews undertaken for accepted manuscripts. Reviewers are asked to complete a checklist review form and are provided with free text boxes to elaborate on their assessment. These free text comments are reproduced below.

ARTICLE DETAILS

Title (Provisional)

Implementing a bundle of interventions to support older adults transitioning from hospital to residential aged care: a protocol for the process evaluation of the OPTIMAL stepped wedge cluster randomised controlled trial

Authors

Fernando, Rangika L; Crotty, Maria; Inacio, Maria C; Batta, Ishita; Bourke, Alice; Forward, John; Furst, Chloe; Whitehead, Craig; Shaw, Sandra; Shepperd, Luke; Harvey, Gillian

---

VERSION 1 - REVIEW

**Reviewer**	**1**
**Name**	**Schols, Jos**
**Affiliation**	**Maastricht University, Department Health Services Research, School for Public Health and Primary Care (CAPHRI)**
**Date**	**05-Dec-2025**
**COI**	**None**

This is a well written article which is complementary to another protocol article as mentioned in ref. 24.

Some minor remarks:

I miss figure 2 with the timeline and exact dates. The dates of the study should be

included in the manuscript.

In the discussion I would like to see some remarks about to what extent the study process contributes to getting the desired effects of the intervention.

The authors state: "It is anticipated that the process evaluation will contribute to a better understanding of the overall process of implementation, scalability and transferability of the transitional care intervention in the Australian context".

Can they also incorporate and discuss useful implementation recommendations for adoption and impelementation of this intervention in a more international context as well?.

VERSION 1 - AUTHOR RESPONSE

Reviewer 1 comments:

5. This is a well written article which is complementary to another protocol article as mentioned in ref. 24.

Thank you, we greatly appreciate your feedback on our manuscript.

Some minor remarks:
6. I miss figure 2 with the timeline and exact dates. The dates of the study should be included in the manuscript.

Thank you for this feedback, we have included the timeline and exact dates in Figure 2 of this manuscript. The manuscript has been updated to include the dates of the study:

Lines 304-305

The process evaluation will be conducted over the same 14 months, and for up to three months post-intervention (i.e. 1st September 2024 to 31st January 2026).

7. In the discussion I would like to see some remarks about to what extent the study process contributes to getting the desired effects of the intervention.

Thank you for this feedback. The process evaluation seeks to understand the process of implementing the OPTIMAL intervention, including any contextual facilitators and barriers, and their effects on the health service outcomes of 30- and 90- day emergency department presentations and readmissions. Once data analysis is completed at the end of the study, it will provide valuable information to inform transferability and scalability of the OPTIMAL intervention.

During the OPTIMAL trial, the process evaluation may directly contribute to achieving the desired effects of the intervention through implementation facilitation. Relevant findings that emerge during the ongoing analysis of data, such as those related to modifiable barriers and enablers to implementation, will be discussed within the central project team, and where necessary, shared as feedback to the locally appointed nurse facilitator to enhance their responsiveness to characteristics of the innovation, recipients and context during implementation.

We have updated the manuscript accordingly:

Lines 505-514

The process evaluation seeks to understand the process of implementing the OPTIMAL intervention, including any contextual facilitators and barriers, and their effects on the

health service outcomes of 30- and 90- day emergency department presentations and readmissions. During the OPTIMAL trial, the process evaluation may directly contribute to achieving the desired effects of the intervention through implementation facilitation. Relevant findings that emerge during the ongoing analysis of data, such as those related to modifiable barriers and enablers to implementation, will be discussed within the central project team, and where necessary, shared as feedback to the locally appointed nurse facilitator to enhance their responsiveness to characteristics of the innovation, recipients and context during implementation.

8. The authors state: "It is anticipated that the process evaluation will contribute to a better understanding of the overall process of implementation, scalability and transferability of the transitional care intervention in the Australian context". Can they also incorporate and discuss useful implementation recommendations for adoption and implementation of this intervention in a more international context as well?

Thank you for the feedback, and certainly, it is envisaged that the process evaluation will contribute to useful implementation recommendations in a more international context as well. We have updated the manuscript accordingly:

Lines 515-517

It is anticipated that the process evaluation will contribute to a better understanding of overall process of implementation, scalability and transferability of the transitional care intervention in both the Australian context, and internationally.

Thank you for your consideration, and please let us know if any further clarifications or revisions are required.

Thank you.

Kind regards,

Rangika L. Fernando

References

1. Fernando RL, Karnon J, Crotty M, et al. Optimising older People's Transition from acute care Into residential aged care through Multidisciplinary Assessment and Liaison (OPTIMAL): protocol for a stepped wedge cluster randomised controlled

trial with embedded process evaluation. *BMC Geriatrics* 2025;25(1):550. doi: 10.1186/s12877-025-06187-y
2. Kitson A, Harvey G, McCormack B. Enabling the implementation of evidence based practice: a conceptual framework. *Qual Health Care* 1998;7(3):149-58. doi: 10.1136/qshc.7.3.149
3. Harvey G, Kitson A. PARIHS revisited: from heuristic to integrated framework for the successful implementation of knowledge into practice. *Implementation Science* 2016;11(1):33. doi: 10.1186/s13012-016-0398-2
4. Hunter SC, Kim B, Kitson AL. Mobilising Implementation of i-PARIHS (Mi-PARIHS): development of a facilitation planning tool to accompany the Integrated Promoting Action on Research Implementation in Health Services framework. *Implementation Science Communications* 2023;4(1):2. doi: 10.1186/s43058-022-00379-y
